# Quercetin as a JAK–STAT inhibitor: a potential role in solid tumors and neurodegenerative diseases

**DOI:** 10.1186/s11658-022-00355-3

**Published:** 2022-07-26

**Authors:** Hamidreza Zalpoor, Mohsen Nabi-Afjadi, Razieh Forghaniesfidvajani, Chanour Tavakol, Faranak Farahighasreaboonasr, Farid Pakizeh, Vahid Ghobadi Dana, Farhad Seif

**Affiliations:** 1 Shiraz Neuroscience Research Center, Shiraz University of Medical Sciences, Shiraz, Iran; 2 Network of Immunity in Infection, Malignancy & Autoimmunity (NIIMA), Universal Scientific Education & Research Network (USERN), Tehran, Iran; 3 Department of Biochemistry, Faculty of Biological Science, Tarbiat Modares University, Tehran, Iran; 4 Tehran University of Medical Sciences, Tehran, Iran; 5 Department of Biology, Zand Institute of Higher Education, Shiraz, Iran; 6 Students Research Committee, Tabriz University of Medical Sciences, Tabriz, Iran; 7 Department of Immunology and Allergy, Academic Center for Education, Culture, and Research (ACECR), Tehran, Iran; 8 Neuroscience Research Center, Iran University of Medical Sciences, Enghelab St., Aboureyhan St., Vahid Nazari Crossroad, P17, Tehran, Postal code: 1315795613 Iran

**Keywords:** Quercetin, JAK–STAT inhibitor, Solid tumors, Neurodegenerative diseases, Cancers

## Abstract

The Janus kinase–signal transducer and activator of transcription (JAK–STAT) pathway is involved in many immunological processes, including cell growth, proliferation, differentiation, apoptosis, and inflammatory responses. Some of these processes can contribute to cancer progression and neurodegeneration. Owing to the complexity of this pathway and its potential crosstalk with alternative pathways, monotherapy as targeted therapy has usually limited long-term efficacy. Currently, the majority of JAK–STAT-targeting drugs are still at preclinical stages. Meanwhile, a variety of plant polyphenols, especially quercetin, exert their inhibitory effects on the JAK–STAT pathway through known and unknown mechanisms. Quercetin has shown prominent inhibitory effects on the JAK–STAT pathway in terms of anti-inflammatory and antitumor activity, as well as control of neurodegenerative diseases. This review discusses the pharmacological effects of quercetin on the JAK–STAT signaling pathway in solid tumors and neurodegenerative diseases.

## Introduction

### Quercetin

Given their highly potent therapeutic activity, fewer side effects, and cost-effectiveness, natural products may be promising candidates for pharmaceutical development as anticancer, anti-autoimmunity, and anti-allergic agents. There is a family of natural compounds called flavonoids that are found widely in flowers and fruits in the form of benzo-gamma-pyrone derivatives. Quercetin is one of these natural products [1]. Quercetin (3,3′,4′,5,7-pentahydroxyflavone) represents the most abundant flavonoid in human diets and can be found at high concentrations in apples, onions, red wine, red grapes, tea (*Camelia sinensis*), capers, broccoli, lovage, and a wide range of berries [2, 3]. Various cancer cell types, including liver cancer, have been shown to be affected by antineoplastic activity of quercetin via its influence on proliferation, differentiation, and apoptosis [4]. Additionally, quercetin downregulates multiple signal transduction pathways, including NF-κB, MEK–ERK, PI3K–Akt–mTOR, Wnt–β-catenin, and Nrf2–keap1, which contribute to inflammation and carcinogenesis processes [5–7]. However, the pleiotropic properties of quercetin warrant further study of other proteins that might also be involved in its mechanisms of action.

### JAK–STAT signaling pathway

The Janus kinase–signal transducer and activator of transcription (JAK–STAT) pathway exerts an important role in transducing signals from cell membrane receptors to the nucleus [8, 9]. The JAK family of tyrosine kinases bind to the cytoplasmic regions of type I and type II cytokine receptors. The binding of ligands to receptors results in the multimerization of receptors. Some receptor subunits are expressed as homodimers, such as growth hormone and erythropoietin, while others are expressed as heteromultimers such as interleukins (ILs) and interferons (IFNs). Receptors associated with JAKs are activated to start transphosphorylation of JAKs and subsequent recruitment of STATs. Human JAK family has four members: JAK1, JAK2, JAK3, and TYK2 [8, 10]. JAK family members have several distinct domains, including N-terminal FERM domain, Src homology 2 (SH2) domain, pseudokinase domain, and conserved PTK domain [8]. STAT is one of the most prominent transcriptional factor families in cancers, consisting of seven structurally similar members: STAT1, STAT2, STAT3, STAT4, STAT5a, STAT5b, and STAT6 [11, 12]. Generally, STAT family members contain six common functional domains: a coiled-coil domain (CCD), an N-terminal domain (NH2), a DNA-binding domain (DBD), an SRC homology 2 domain (SH2), a linker domain, and a transactivation domain (TAD). Aberrant activation of the JAK–STAT pathway is evident in various diseases such as cancers [13] and neurodegenerative diseases [14]. In many cancers, STAT3 is constitutively activated. There are multiple original studies on this issue investigating the role of STAT3 signaling in several solid tumors [13].

### Quercetin properties: structure and biologic functions

Quercetin, also known as 2,3,4,5,7-pentahydroxyflavone or 2,3,4,5,7-dihydroxyphenyl-3,5,7-trihydroxychromen-4-one, contains two benzene rings, A and B, that are connected by a pyrone ring. Owing to the presence of two antioxidant pharmacophores in quercetin’s structure, it can effectively neutralize free radicals and join transitional metal ions. Furthermore, catechol, in conjunction with the OH group present at position C3 in the structure of quercetin, is a highly effective way to scavenge free radicals. As a pentalhydroxyl flavonol, hydroxyl groups of quercetin are positioned on the 3rd, 30th, 40th, and 7th carbon positions in the flavonol structure. Interestingly, with the replacement of diverse functional groups, quercetin becomes more biochemically and pharmacologically active [15–17]. Some studies suggest that quercetin is present in two forms: as a standalone compound and as an aglycone or as a mixture with other compounds. In addition to carbohydrates, lipids, alcohols, and sulfate groups, it can be reacted with other molecules to produce numerous derivatives of quercetin, including glycosides, ethers, prenylated quercetins, and sulfated derivatives [17]. Quercetin glycoside is the most abundant flavonoid compound in propolis as well as many other dietary supplements, including fruits and vegetables, such as onions, broccoli, apple, tea, and red wine [15]. Aside from acting as an anticancer, antitumor, anti-ulcer, anti-allergy, antiviral, anti-inflammatory, and antidiabetes agent, it also exerts gastroprotection, antihypertension, immune modulation, and anti-infection effects [18]. A preclinical study demonstrated that quercetin significantly reduced levels of inflammation moderators, including NO synthase, COX-2, and CRP, in a human hepatocyte-derived cell line [19]. Studies conducted on rats have shown that quercetin (80 mg equivalent dose) inhibits acute and chronic inflammation and that it also has significant anti-arthritic properties against adjuvant-induced arthritis [20, 21]. This bioactive compound has also been studied for its anticancer properties. Quercetin is believed to inhibit cancers of various solid tissues, such as breast, lung, nasopharyngeal, kidney, colorectal, prostate, pancreatic, and ovarian cancers, as well as neurodegenerative diseases [15]. In this regard, quercetin is not harmful to healthy cells, while it can impose cytotoxic effects on cancer cells through a variety of mechanisms, making it an effective supplementary agent for treating solid tumors and neurodegenerative diseases in combination with other anticancer medications [22]. Despite the range of biological benefits mentioned above, quercetin has been restricted in its pharmaceutical application as a result of its low hydrosolubility, lack of stability in physiology, high metabolism in the liver prior to reaching the bloodstream, and low bioavailability [23, 24].

In patients with cancer, quercetin was intravenously injected at a dose of 60–2000 mg/m^2^. The researchers determined a safe dose to be 945 mg/m^2^. A toxic dose resulted in vomiting, high blood pressure, nephrotoxicity, and a decrease in serum potassium. In intravenous administration, quercetin has a half-life of 0.7–7.8 min. Dispensation content is 3.7 L/m^2^, and elution is 0.84 L/min/m^2^ [25]. At a dose of 200 mg, Graefe and colleagues investigated the pharmacokinetics of quercetin. Quercetin has been reported to have a *C*_max_ and a *T*_max_ of 2.3–1.5 g/mL and 0.7–0.3 h, respectively [26]. According to these observations, numerous quercetin analogs and nanoconjugates of quercetin containing various delivery systems have been demonstrated, including liposomes, silver nanoparticles, poly lactic-co-glycolic acid (PLGA), and polymeric micelles, for example, DOX-conjugated micelles, metal-conjugated micelles, nucleic-acid-conjugated micelles, and antibody-conjugated micelles, which show a variety of biological implications such as biphasic, inotropic impacts [27, 28]. For instance, Iacopetta et al. synthesized a series of quercetin derivatives referred to as Q2–Q9 using catechol ketal as the starting material in which the OH groups were all or partially replaced with hydrophobic functional groups such as acetyl esters, ethyl or benzyl ethers, or diphenyl ketals of the catechol system. By inhibiting topoisomerases I and II and modulating intracellular reactive oxygen species (ROS) production, these chemical modifications produced compounds with higher anticancer activity relative to the canonical quercetin in two breast cancer models. Accordingly, quercetin exerts its antioxidant effects via competitive inhibition of the enzyme xanthine oxidase and noncompetitive inhibition of the enzyme xanthine dehydrogenase [29].

In addition, a study by Guan and colleagues prepared and evaluated quercetin nanoparticles (QPTN) as a means of improving the solubility of quercetin with PLGA and TPGS. The results demonstrated that QPTN was capable of inducing HepG2 cell apoptosis in a dose-dependent way and suppressing tumor growth by 59.07% [30].

### JAK–STAT inhibitory effects of quercetin on cancer

**Fig. 1:**
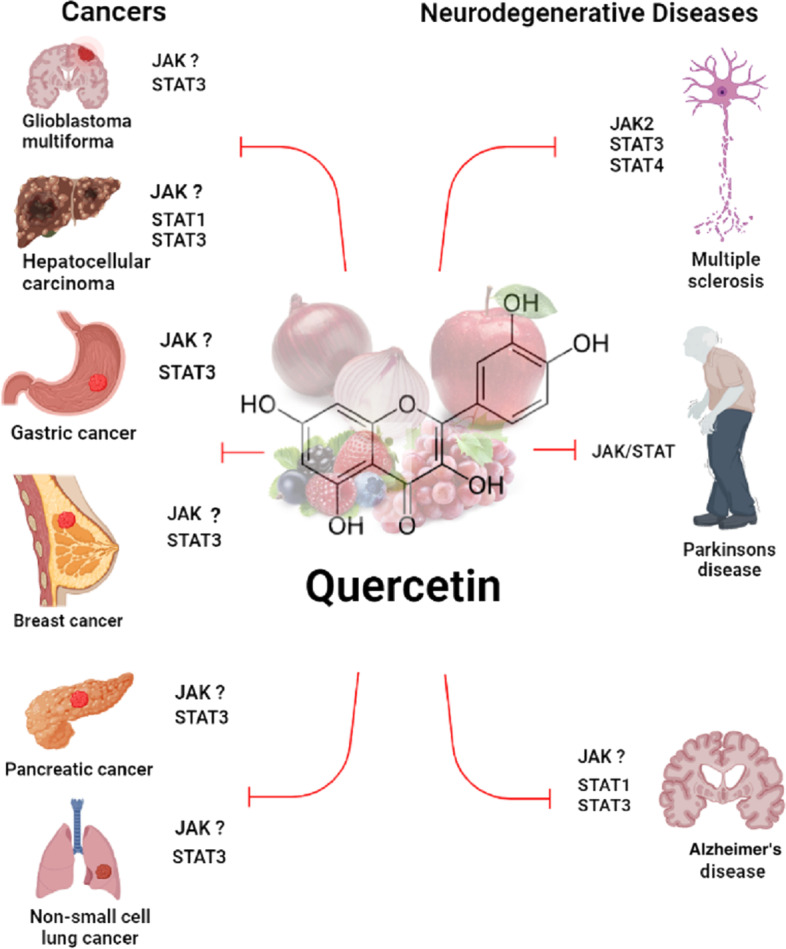
Therapeutic effects of quercetin on inhibiting the JAK–STAT signaling pathways in cancers and neurodegenerative diseases

The specific effect of quercetin on tumor cells without any impact on normal or nontransformed cells has compelled many researchers to explore its potential use as an adjuvant in suppressing proliferation, metastasis, epithelial–mesenchymal transition (EMT), and oxidative stress [31–33]. Having more potent antitumor activity, fewer side effects, and cost-effectiveness, natural products are promising candidates for pharmaceutical development as anticancer agents. Recently, studies reported that the expression of antiviral genes, such as 2′-5′-oligoadenylate synthetase (2′5′-OAS) and RNA-activated protein kinase (PKR), are stimulated by type-I IFNs through the JAK–STAT pathway [34–36]. Although the exact mechanism of action on the JAK–STAT pathway remains unclear, tofacitinib and ruxolitinib are JAK inhibitors that have been approved by the Food and Drug Administration (FDA) for their inhibitory effect on some lymphoma tumor cells. High doses of these drugs cause side effects despite their “pan-JAK” inhibitory effects, including infections and malignant tumors, gastrointestinal perforation, venous thromboembolism, dyslipidemia, and other complications [37]. Patients with rheumatoid arthritis taking tofacitinib, the first JAK inhibitor on the market, have suffered pulmonary thrombosis and death due to high doses of the drug. On the other hand, studies have shown that quercetin increased type-I IFN-induced JAK–STAT signaling through the inhibition of SHP2. It was reported that quercetin has a synergistic effect when concomitantly using some anticancer drugs [12]. According to Tiwari et al., an investigation compared quercetin and gefitinib loaded onto polyvinylpyrrolidone (PVP)-functionalized graphene oxide (GO-PVP) with quercetin and gefitinib separated by polyvinylpyrrolidone (PVP)-functionalized graphene oxide. The combined drugs loaded onto the GO-PVP nano platform were significantly more toxic than the individual drugs loaded onto the platform, as well as the free drugs, in regard to PA-1 cells [38]. Here, we described antitumor effects of quercetin by targeting JAK–STAT signaling pathways (Fig. 1).

Moreover, we suggest that more investigations based on natural products are needed to provide a promising therapeutic approach to enhance the efficacy of anticancer therapy (e.g., chemotherapy, radiotherapy, or radiochemotherapy) in a less toxic manner, also before or after tumor surgery.

### Glioblastoma multiforme

Glioblastoma multiforme (GBM) is a malignant tumor of connective tissue, and has been known as the most deadly primary brain tumor [1, 39–41]. Flavonoids such as quercetin are able to cross the blood–brain barrier (BBB) via different mechanisms, including carrier-mediated transcellular transport transcellular diffusion or paracellular distribution via tight junctions between BBB endothelial cells [1, 42]. In gliomagenesis, *STAT3* may act as an oncogene [43]. Consequently, inflammatory microenvironments may favor tumor development [44]. A potential role for quercetin in the prevention and treatment of glioblastoma has been demonstrated as a suppressor of the STAT3 activation signaling pathway stimulated by IL-6 [44, 45]. In this line, IL-6-induced JAK–STAT3, Rb phosphorylation, cyclin D1 expression, and MMP-2 secretion are inhibited by 48 h treatment with 25 µM quercetin in T98G and U87 GBM cell lines [44].

On the other hand, mouse peritoneal macrophages were isolated and quercetin was used to treat the ex vivo inflammation induced by lipopolysaccharide in a study by Liao et al. There was a significant decrease in TNF-α*TNF-α* gene expression levels as well as pro-/anti-inflammatory ratios (TNF-α/IL-10) and inactivation of inflammatory macrophages, but *IL-10* gene expression levels were elevated in quercetin-treated macrophages. This suggests that quercetin treatment has overall anti-inflammatory potential. In the activated inflammatory macrophages, quercetin inhibited *TLR2* gene expression as well as STAT3 protein phosphorylation [46]. Moreover, as a potential prognostic marker for glioblastoma multiforme (GBM), Axl plays a crucial role. Signals downstream of it include those from NF-κB, JAK–STAT, and PI3K–Akt–mTOR [47]. With 0, 25, 50, and 100 µM doses of quercetin, dose-dependent apoptosis was observed in two GBM cell lines, U87MG and U373MG, resulting from decreased Axl protein expression, IL-6 expression levels, and STAT3 phosphorylation [48]. Furthermore, it was found that quercetin inhibited glioblastoma cell invasion and angiogenesis in vitro. It is possible that quercetin inhibits migration and angiogenesis via downregulating the levels of VEGFA, MMP9, and MMP2 [49].

Moreover, a previously conducted study synthesized an anticancer drug commonly used in treating glioblastoma that combined nanoquercetin (nanohydrogels) with temozolomide to enhance its specificity and efficacy. As a codelivery vehicle for temozolomide and quercetin, the nanohydrogel facilitated the internalization and cytotoxicity of quercetin. In addition, using quercetin-containing nanohydrogels significantly reduced IL-8 production, IL-6 production, and VEGF production in a dose-dependent manner in pro-inflammatory conditions, with meaningful implications for glioblastoma cells’ drug resistance. However, it was used at 100 and 200 µg doses [50].

Recent evidence suggests that supplementation of standard therapy with quercetin enhances the efficacy of treatment of experimental glioblastoma by inducing apoptosis through caspase-3 and PARP-1 cleavage, and suppressing PI3K–Akt activation. When combining quercetin and irradiation, the breakdown of caspase-3, PARP-1, phosphorylated ERK, JNK, p38, and RAF1 were increased, whereas phosphorylated Akt was considerably decreased. By enhancing apoptosis through inducing caspase-3 and PARP-1 cleavage and suppressing activation of the Akt pathway, quercetin supplements standard therapy with improved efficacy in treating experimental glioblastoma [51].

Quercetin also induces apoptosis in human glioblastoma multiform T98G cells, which is associated with activation of caspase 3 and 9 and release of cytochrome C from the mitochondria. It has been suggested that apoptosis is initiated by stress in the endoplasmic reticulum by increasing expression of caspase 12 and the presence of multiple granules in the cytoplasm upon temozolomide treatment with or without quercetin [52].

### Hepatocellular carcinoma

Hepatocellular carcinoma (HCC), one of the most common cancers worldwide, is the second and sixth leading cause of cancer-related death in men and women, respectively [53]. It is estimated that 600,000–800,000 new cases of HCC occur annually on a global level [54]. Various intracellular signaling pathways activation implicated in cell growth, survival, apoptosis, angiogenesis, and differentiation have been found to facilitate HCC development and progression. These oncogenic signaling pathways include JAK–STAT, PI3K–Akt–mTOR, Wnt–β-catenin, and Ras–Raf–MAPK pathways [55]. According to recent investigations, the signal transducer and activator of transcription protein 1 (STAT1) was phosphorylated by quercetin, promoting endogenous IFN-α-regulated gene expression. Additionally, quercetin reduced the proliferation rate of hepatocellular carcinoma HepG2 and Huh7 cells when IFN-α expression was stimulated [6]. In the development of HCC, STAT3 is generally accepted as an oncogene. Activation of STAT3 stimulates the expression of several genes that play a significant role in cancer development, emphasizing the importance of STAT3 in HCC [54]. Wu et al.’s study revealed that quercetin suppresses hepatocellular carcinoma progression via modulating cellular invasion, migration, and autophagy. Moreover, its effect may partly be attributed to the downregulation of the JAK2–STAT3 signaling pathway [56]. Also, quercetin plays a suppressive role against HCC cells through apoptosis and p16-mediated cell cycle arrest, and its combination with cisplatin displayed synergistic inhibitory activity in triggering apoptosis and suppressing cancer cell growth [57]. A microprecipitation high-pressure homogenization technique, with methyl polyethylene glycol-Deoxycholic acid (mPEG-DCA) as a stabilizer, was used to synthesize three quercetin nanosuspensions containing different particle sizes. All three quercetin nanoparticles (QUR-NPs) were found to have significantly stronger inhibitory activity against MCF-7 cells and hepatocyte 7702 cells in vitro, while inhibiting tumors against the murine hepatic carcinoma H22 model in vivo [58].

The effects of quercetin on in vivo and in vitro hepatocellular carcinoma progression were also examined in a study using LM3 and nude mouse tumor models, in which quercetin suppressed cell apoptosis, migration, invasion, and autophagy by inhibiting the JAK2–STAT3 signaling pathway [56].

### Gastric cancer

Despite declines in incidence rates, increased awareness, and advances in treatment strategies, gastric cancer remains one of the most prevalent forms of cancer worldwide [59]. The development and progression of gastric cancer are influenced by *Helicobacter pylori* infection, lifestyle factors, dietary factors, and various genetic aberrations [60, 61]. Upon entry into human gastric epithelial cells, the CagA protein of *H. pylori* may exhibit tyrosine phosphorylation with downstream effects on signal transduction. Researchers have found that the CagA phosphorylation status affects the signal switch between the JAK–STAT3 and SHP2–ERK pathways via gp130, elucidating a novel mechanism for the *H. pylori* signaling pathway [62, 63].

Furthermore, González-Segovia et al. found that oral quercetin administration reduced *H. pylori* infection and both inflammatory response and lipid peroxidation in vivo [64]. Quercetin inhibits the proliferation of human gastric cancer (GC) MGC-803 cells. This may be related to the downregulation of leptin and leptin receptor protein, leptin mRNA, and leptin receptor mRNA expression by the JAK–STAT pathway [65]. Also, combined treatment with quercetin and curcumin was observed with substantial inhibition of cell proliferation, associated with loss of mitochondrial membrane potential (Δ*Ψ*_m_), cytochrome C release, and decreased ERK and AKT phosphorylation. These findings suggest that the combination of quercetin and curcumin leads to apoptosis through the mitochondrial pathway [66].

### Breast cancer

Women worldwide are most likely to develop breast cancer with a high rate of mortality [67, 68]. Despite improvements in chemotherapeutic agents, new strategies to overcome tumor cell survival remain elusive despite progress in understanding the mechanisms of chemoresistance [69, 70].

To create an ideal cancer treatment protocol, we need to identify key target molecules, as well as safe and stable delivery systems that will allow us to overcome resistance and minimize the side effects of chemotherapeutic agents [71, 72].

In this regard, Safi et al. indicated that concomitantly using docetaxel and quercetin synergistically results in inhibition of both cell growth and cell survival, as well as apoptosis induction in MDA-MB-231 human breast cancer cell line [12]. The results showed that single-agent treatment with docetaxel or quercetin leads to a decrease in the viability of the MDA-MB-231 cells at 48 h, whereas the combination of docetaxel (7 nM) and quercetin (95 μM) displayed the greatest synergistic effects with a combination index value of 0.76 accompanied by enhancing expression of the *p53* gene as well as pro-apoptotic protein BAX, and reducing the expression of STAT3 anti-apoptotic protein BCL2, phosphorylated AKT, and ERK1/2 [12]. Further, quercetin has been revealed to have a role in antiproliferation of HER2-overexpressing BT-474 BC cells via STAT3 signaling suppression and caspase-dependent extrinsic apoptosis activation, while causing an increase in sub-G0/G1 apoptotic population [73].

### Pancreatic cancer

Pancreatic cancer (PC), a highly fatal disease, is one of the most commonly occurring digestive malignancies. PC is one of the leading causes of cancer-related death worldwide [53, 74, 75]. Moreover, patients with PC have a 5-year survival rate of 21.3% for local-stage cancer, 8.9% for regional-stage cancer, and 1.8% for distant-stage cancer [76].

According to Yu et al., quercetin inhibited PATU-8988 and PANC-1 cell growth and reduced MMP release. In this study, they examined whether quercetin treatment affected malignancy in cells by activating STAT3 and IL-6. They indicated that, when MMP secretion and EMT occur, STAT-3 signaling is stimulated, and quercetin reverses IL-6-induced EMT and invasion. It has been revealed that quercetin can inhibit EMT, invasion, and metastasis, as well as reverse the IL-6-induced increase in PC cells through inhibiting the STAT3 signaling pathway [77]. Pang et al. found that quercetin hindered PC death by improving fatty acid uptake, enhancing cell adhesion, stimulating the immune system, and increasing thrombospondin-1 activity [78]. Additionally, previous research has indicated that quercetin inhibits BCL-2 protein synthesis and upregulates the *p53* gene, suggesting that it has pro-apoptotic effects. However, inhibiting BCL-2 transcription may prevent tumor growth [79]. Nwaeburu et al. found that quercetin treatment induced the expression of microRNA 200b-3p in pancreatic cancer AsPC1 cell lines, which appears to be crucial in regulating Notch signaling and irregular division of pancreatic ductal adenocarcinoma (PDA) cells [80]. Furthermore, it has been found that quercetin suppressed the nuclear translocation and phosphorylation of Smad2 and Smad3. When downstream signaling is stimulated by TGF-β1, Smad2 and Smad3 form heteromeric complexes with Smad4 and translocate to the nucleus, inducing the expression of EMT-inducing transcription factors (EMT-TFs) [32].

### Non-small-cell lung cancer

One of the leading causes of cancer-related death worldwide is non-small-cell lung cancer (NSCLC) [81]. Lung carcinoma can be divided into two subtypes on the basis of morphological and genetic features: non-small-cell lung cancer (NSCLC) and small-cell lung cancer (SCLC) [82, 83]. NSCLC accounts for 80–85% of all lung carcinoma cases [84, 85].

Several molecules, including flavonoids, have been found to be beneficial in treating NSCLC. Several cancers have already been reported to be prevented by quercetin, including lung cancer [86, 87]. According to Mukherjee et al.’s study, in NSCLC with constitutive IL6–STAT3 activation for uncontrolled cell proliferation, targeted blockade of NF-κB and IL-6–STAT3 signaling by quercetin (66 µM of quercetin for 12, 18, 24, 36, and 48 h) represents an innovative approach for treating NSCLC. Through inhibition of IL-6–STAT3 signaling pathways, particularly those involved in NF-κB activation, the Bcl2–Bax imbalance can then trigger the cancer cell to undergo self-destruction by triggering apoptosis [87]. These observations may provide valuable information for the potential application of quercetin in NSCLC prevention/therapy.

### Other cancers

In recent years, there have been numerous developments regarding the JAK–STAT inhibitory effects of quercetin in other types of cancer. For instance, compared with docetaxel alone, quercetin increased inhibition of PI3K–Akt and the STAT3 signaling pathways in androgen-dependent prostate cancer cells, and decreased expression of the multidrug-resistance-related protein. At a concentration of 5 µM, quercetin also significantly enhanced cell cycle arrest at G2/M phase and sensitivity to chemotherapy in LAPC-4-AI and PC-3 prostate cancer cells [88–91].

Moreover, the pretreatment of ovarian cancer with quercetin significantly increased the cytotoxicity of cisplatin and activated the stress response of the three branches of the endoplasmic reticulum [92]. Further, it inhibited STAT3, resulting in the downregulation of the *BCL-2* gene downstream of STAT3, and improved the antitumor effect of cisplatin in a mouse xenograft model for ovarian cancer [93–95].

In response to EGCG, STAT1 and STAT3 phosphorylation was reduced dose-dependently. Quercetin and EGCG was synergistic and inhibited cells’ upregulation of inducible nitric oxide synthase (iNOS) and intercellular adhesion molecule-1 (ICAM-1) in response to cytokine-induced iNOS and ICAM-1 levels induced by JAK–STAT activation in cholangiocarcinoma cells. According to recent studies on primary effusion lymphoma (PEL) cells, quercetin inhibits the STAT3 and PI3K/Akt/mTOR pathways, thereby downregulating prosurvival cellular proteins, including cMyc and cyclin D1. Furthermore, quercetin decreased the IL-6 and IL-10 release, resulting in PEL cell death [96]. However, besides numerous studies on anticancer effects of quercetin, there is an information gap on the JAK–STAT inhibitory effects on other cancer types such as colorectal, skin, kidney, thyroid, eye, cervical, blood, and bone cancers that should be investigated.

## JAK–STAT inhibitory effects of quercetin on neurodegenerative diseases

There are a number of neurodegenerative diseases, including Alzheimer’s disease (AD), Parkinson’s disease (PD), multiple sclerosis (MS), leukodystrophies, and diseases involving neuron and/or glia degeneration [97]. Even though the central nervous system (CNS) was once considered immune-privileged, nowadays we know that T cells continuously patrol the CNS and innate immunity is the first line of defense for the CNS. Demyelination and/or degeneration of neurons are caused by aberrant activation of innate immune cells, which releases pro-inflammatory cytokines, chemokines, ROS, and NO, or polarizes and activates effector T cells and myeloid cells and T cells. The majority of these processes are dependent on JAK–STAT signaling pathways, and a number of neurodegenerative diseases are characterized by inflammation in the CNS, and the JAK–STAT signaling pathway leads to pathogenic inflammation [14]. In this line, by regulating STAT1 signaling, T-helper type 1 (Th1) cells produce cytokines that alter the balance between Th1 and Th2 cells, altering immune function and inflammation. On the other hand, as a result of STAT1 hyperactivation and defective nuclear dephosphorylation, mutations in the *STAT1* gene trigger chronic mucocutaneous candidiasis and adversely affect Th1 and Th17 cell responses [98].

Hence, using natural compounds such as quercetin alone or in combination with other drugs could be used to treat neurodegenerative diseases by decreasing neuroinflammation through the JAK–STAT signaling pathway (Fig. 1). For example, according to a study carried out on C6 glioma cells, nanoliposomes can increase quercetin anticancer activity by inhibiting the JAK2–STAT3 pathway and mitochondrial ROS generation [99]. Additionally, coupling quercetin with nanoparticles, such as β-cyclodextrin-dodecylcarbonate, makes it more readily permeable through the blood–brain barrier, making it an excellent candidate for STAT intervention and neuroinflammatory treatment [99].

### Multiple sclerosis

Multiple sclerosis (MS), a chronic neurodegenerative and demyelinating disease that impacts the CNS, is characterized by aggressive lesions throughout the brain and spinal cord. MS affects approximately 2.5 million people worldwide. Affected people are typically between the ages of 20 and 40 years, and the disease prevalence is three times higher among women than among men [100–102]. Mirzazadeh et al. studied the effects of quercetin administration (10 mg/kg daily) on the development of disability in rat models of multiple sclerosis. Quercetin therapy led to a better outcome in rats. There was a significant increase in myeloperoxidase activity and nitric oxide levels in the serum of the rat models that were treated with quercetin, as well as in the level of lipid peroxidation in the brain tissue [103]. As documented in another study, quercetin (25 or 50 mg/kg daily for 7 or 14 days) reduced the latency of visual evoked potential (VEP) waves in rats with lysolecithin (LPC)-induced demyelination when ingested orally. In addition, quercetin treatment decreased glial activation in treated animals compared with the control group. Moreover, following LPC injection, quercetin treatment reduced demyelinated areas and led to an increase in remyelination [104].

According to Muthian et al., by inhibiting IL-12-induced tyrosine phosphorylation of STAT3, STAT4, JAK2, and TYK2, quercetin inhibits the proliferation of T cells and differentiation of Th1. Muthian et al. in this in vitro study suggested that quercetin may be useful in treating MS and other autoimmune diseases caused by Th1 cells by inhibiting IL-12 signaling and Th1 differentiation [105]. In addition, in a recent study, SJL/J mice were treated every other day for 25 days with 50 or 100 µg of quercetin during an experimental autoimmune encephalomyelitis model. Quercetin reduced inflammation in mice compared with control and quercetin-treated mice on many parameters, such as pathological score (CNS demyelination and inflammation), immune system responses toward neural antigens and IL-12, production of splenocytes, macrophages, and microglia, and activation of the JAK–STAT pathway [105].

### Parkinson’s disease

Parkinson’s disease (PD) is a neurodegenerative disease characterized by neuroinflammation, oxidative stress, and selective dopaminergic neuronal loss. Therefore, available pharmacotherapy is based on supplying dopamine precursors or on stimulation of dopamine synthesis in the remaining neurons and modulating neuroinflammation and oxidative stress [106]. In comparison with PD-induced controls, quercetin 30 mg/kg was administered for 14 consecutive days to 6-OHDA-intoxicated rats to significantly improve dopamine and antioxidant enzyme levels. Furthermore, the quercetin-treated group exhibited fewer dead neurons. In another study, quercetin doses of 100 and 200 mg/kg were significantly effective in alleviating motor balance and coordination in mice with 1-methyl-4-phenyl-1,2,3,6-tetrahydropyridine (MPTP)-induced parkinsonism in the rotarod test [107]. On the other hand, administration of 50 mg/kg of quercetin in rotenone-induced PD in rats led to an improvement in movement, dopamine level, and oxidative balance [108].

In addition to antioxidant properties, quercetin has anti-inflammatory and neuroprotective roles in PD [109]. Many neurodegenerative disorders, including Parkinson’s disease, are characterized by aberrant activation or phosphorylation of the JAK–STAT signaling pathway. In PD, interferon-gamma (IFN-γ) and IL-6 were found to be two of the most potent activators of the JAK–STAT pathway [110]. Therefore, dysregulation of the JAK–STAT in PD and its involvement in various inflammatory pathways make it a promising PD therapy approach. Accordingly, administration of quercetin with piperine alone and in combination significantly prevented neuroinflammation via reducing the levels of IL-6, TNF-α (two potent activators of the JAK–STAT pathway), and IL-1β in PD in experimental rats [109].

### Alzheimer’s disease

Alzheimer’s disease (AD) is typically caused by the accumulation of amyloid-β (Aβ) aggregates and hyperphosphorylation of tau proteins, resulting in synaptic dysfunction and neurofibrillary tangles (NFTs). AD accounts for 60–80% of total dementia cases, and it mostly affects the elderly (65 years old or older). AD affects about 35.6 million people worldwide, with 4.6 million newly diagnosed cases reported every year. Also, from 60 years of age, the prevalence of AD doubles every 5 years [111, 112]. The levels of STAT1 protein have been shown to be elevated in both cytosolic and particulate fractions from the cortex of patients with AD in comparison with healthy controls [113]. In the APPswe/PS1dE9 and 3xTg mouse models of AD, *STAT3* immunoreactivity has been found to increase in the nucleus of GFAP- and vimentin-immunoreactive astrocytes. Lentivirus-mediated expression of suppressor of cytokine signaling protein 3 (*SOCS3*), a negative regulator of this pathway, downregulated STAT and inhibited JAK-specific activity, and suppressed nuclear STAT3 immunoreactivity as well as GFAP immunoreactivity in astrocytes, implicating the JAK–STAT3 pathway in astrocytic reaction [114].

Under certain conditions, Aβ, which is believed to play an important role in this pathology, is neurotoxic. By activating JAK2–STAT3, nicotinic acetylcholine receptors are known to reduce Aβ neurotoxicity, but whether *STAT3* gene regulation is required is unknown [115]. It was found that quercetin suppressed the formation and stability of Aβ fibrils. In this line, multiple steps of the formation of sclerotic plaques were inhibited by quercetin suppressing β-secretase (an enzyme engaged in Aβ formation) and aggregation of Aβ [116, 117].

Moreover, Aβ neurotoxicity is prevented by quercetin and its derivatives via activating the JAK2–STAT3 signaling pathway and maintaining cholinergic activity [118]. Quercetin, is also preclinically reported to be neuroprotective in Alzheimer’s disease because of its antioxidant and anti-inflammatory activity. In neuroinflammation, the release of cytokines, such as TNF-α and IL-1β, by astrocytes and microglia is regulated by triggers such as protein aggregation and neuronal death, initiating an immune response. Cytokine release can be reduced by inhibiting the inducible forms of cyclooxygenase (COX-2) and lipoxygenase (LOX) [119]. Quercetin acts as a pro-antioxidant, inhibiting the release of neuroinflammatory mediators such as interleukin-6 (IL-6), IL-1β, COX-2, LOX, and tumor necrosis factor (TNF-α) by oxidative stress [106, 119].

It was also interesting to find that, in a SAMP8 mouse study, the effects of free quercetin (25 mg/kg/day) and nano-encapsulated quercetin particles (25 mg/kg every 2 days) were evaluated. Almost twofold more quercetin was found in the brains of mice treated with quercetin nanoparticles, which resulted in major effects on memory and learning [120]. Moreover, quercetin acts as a pro-antioxidant, inhibiting the release of neuroinflammatory mediators such as interleukin-6 (IL-6, IL-1β) and tumor necrosis factor (TNF-α) by oxidative stress.

Quercetin is now in phase II of a clinical trial to evaluate its safety and feasibility as a senolytic for Alzheimer’s. Researchers chose to conduct the study after demonstrating that a combination of drugs used in the trial prevented neuron death in a mouse model of AD. Further, quercetin and dasatinib are both known to be senolytics, substances that remove senescent cells in a selective manner, therefore playing a role in many age-related or age-predisposed diseases [121].

## Conclusions and future perspectives

Natural polyphenolic compounds like quercetin that inhibit JAK and STAT family members have been found to have fewer side effects and less toxicity. The anti-inflammatory, antiproliferative, and anti-angiogenic activities of quercetin make it an excellent cancer-prevention agent. Consequently, we suggest that quercetin can be used as an effective, safe, and cost-effective JAK/STAT inhibitor alone or concomitantly with other JAK inhibitors for solid tumors and neurodegenerative disease. More investigations are needed to evaluate potential JAK–STAT inhibitory effects of quercetin for other malignancies, as well as neurodegenerative and autoimmune diseases. Although quercetin may exhibit considerable anticancer activity, it has poor solubility in water, low permeability, and high metabolism in the liver before reaching the bloodstream, resulting in low bioavailability. Moreover, Dajas et al. discovered that quercetin oxidization produces quinones that are not reduced by antioxidants such as tocopherol (vitamin E) and ascorbate (vitamin C), leading to increased damage to neurons (neurotoxicity). The findings of the study suggest that modulating kinases restores redox equilibrium and reduces the discomfort of quercetin restriction [122]. Quercetin should undergo further structural modifications to overcome these limitations in order to be more effective against cancer. Quercetin, for example, can be delivered using nanoconjugated molecules, such as liposomes, silver nanoparticles, polylactic acid, or polymeric micelles, such as DOX-conjugated micelles, metal-conjugated micelles, nucleic-acid-conjugated micelles, or antibody-conjugated micelles. Quercetin-conjugated nanoparticles have been cited for a variety of advantages, including their controlled drug release, retention in tumors, and anticancer properties.

## Data Availability

Not applicable.

## Abbreviations

JAK: Janus kinase
STAT: Signal transducer and activator of transcription
EMT: Epithelial–mesenchymal transition
BBB: Blood–brain barrier
COX-2: Cyclooxygenase-2
IL: Interleukin
IFN: Interferons
VEGF: Vascular endothelial growth factor
MMP: Matrix metalloproteinase
mTOR: Mammalian target of rapamycin
MAPK: Mitogen-activated protein kinase
NF-κB: Nuclear factor kappa-light-chain-enhancer of activated B cells
PI3K: Phosphatidylinositol-3 kinase
PC: Pancreatic cancer
GBM: Glioblastoma multiforme
HCC: Hepatocellular carcinoma
NSCLC: Non-small-cell lung cancer
SCLC: Small-cell lung cancer
AD: Alzheimer’s disease
PD: Parkinson’s disease
MS: Multiple sclerosis
CNS: Central nervous system
